# Intestinal cestodes of chicken are effectively killed by quinoline-rich extract of *Spilanthes acmella*

**DOI:** 10.14202/vetworld.2020.821-826

**Published:** 2020-04-30

**Authors:** Pawi Bawitlung Lalthanpuii, Kholhring Lalchhandama

**Affiliations:** Department of Life Sciences, Pachhunga University College, Aizawl, Mizoram, India

**Keywords:** cestode, helminthiasis, quinoline, *Spilanthes acmella*, tegument

## Abstract

**Background and Aim:**

*Spilanthes acmella* is used for the treatment of intestinal helminth infections in Mizo traditional medicine. In spite of a variety of drugs developed for helminthiases, an entirely safe and absolutely effective drug is still lacking, so much so that infections remain a major problem in human and animal welfare. In this study, we attempted to substantiate *S. acmella* as an anticestodal agent.

**Materials and Methods:**

The aqueous extract of the aerial parts of *S. acmella* was prepared and from it a bioactive fraction was obtained using column chromatography. Chemical analyses were done using thin-layer chromatography (TLC) and gas chromatography–mass spectrometry (GC–MS). Helminth survival test was performed *in vitro* on an intestinal cestode, *Raillietina tetragona*. Structural effects on the cestode were examined under scanning electron microscopy.

**Results:**

From the bioactive fraction of *S. acmella* extract, TLC indicated the presence of an aromatic quinone, which was identified using GC–MS as a quinoline derivative (2,2,4-trimethyl-1,2-dihydroquinoline having a retention time of 24.97 min and chemical formula of C_12_H_15_N). The quinoline-rich fraction showed concentration-dependent activity against *R. tetragona* as that of albendazole. Scanning electron microscopy of the treated cestode revealed classic anthelmintic effects such as tegumental shrinkage and damage of surface organs. The scolex was shrunk, suckers were degenerated with disintegrated spines, and rostellum was completely collapsed. There were severe damages on the tegument and formation of pit-like scars on the proglottids.

**Conclusion:**

The efficacy of *S. acmella* extract and structural damages it caused on the cestode indicates that it is a potential source of anthelmintic agent and that 2,2,4-trimethyl-1,2-dihydroquinoline contributes to its antiparasitic activity.

## Introduction

Generally classified among neglected tropical diseases, helminthiasis is a persistent factor of major problems in human and veterinary health, food security, and socioeconomic conditions. The situation is aggravated by rapid and widespread evolution of drug resistance in virtually all clinically important helminth parasites to all available anthelmintic drugs [1,2]. Drug resistance has caused mass failure in deworming of domesticated animals for which livestock industries are at a peril in many regions [3,4]. There is no foreseeable solution to the dilemma other than the development of novel drugs and alternative strategies. The situation thus begs for urgent and active research for new drug sources [5].

Medicinal plants have been the major source of pharmaceutical drugs and they often offer lead bioactive compounds for drug development. *Spilanthes acmella* Murr. is one of such plants that are well known in traditional medicine and cuisine in different cultures [6]. It is a small perennial herb of the family Asteraceae used as a vegetable, flavoring agent, and remedy for a cohort of health problems. It is most famous for its practical application in dental health because of its analgesic property. Its distinct menthol-like minty flavor is attributed to these gastronomic and dental usages [7]. In addition, it is used in cosmetics for its mild Botox-like effect and hence the name Botox plant. Further, it is also used for the treatment of anemia, cancer, constipation, diuresis, high fever, flatulence, inflammation, liver abscess, peptic ulcer, and ulcer [8]. It is also known to be effective for malarial infections, including falciparum malaria [9]. In Indian medicine, it is used as an aphrodisiac and as a remedy for impotency, articular rheumatism, dysentery, snakebite, and tuberculosis [10]. Studies have validated the anti-inflammatory [11], analgesic, antipyretic [12], antimicrobial, antioxidant [13], and insecticidal activities [14].

The Mizo people of India and Myanmar have used this plant as a common vegetable and generations of their cultivations had produced a unique variety, which is distinct from the type species. Its highly jagged and ribbed leaves and the dome-shaped inflorescence, which is entirely yellow, are the unique botanical features. In Mizo traditional medicine, the aerial parts of the plant are multipurpose therapy for chronic cephalgia, migraine, dysentery, gastritis, oral and dental infections, rheumatism, and stuttering in children. Its pungent odor is employed as an effective insect repellant [15]. The most exclusive use among the Mizo people is for the treatment of intestinal helminthiases. Therefore, it is of crucial importance to try to understand the scientific rationale of such antiparasitic property.

## Materials and Methods

### Ethical approval

The animal experiment in the study was approved by the Institutional Ethics Committee of Pachhunga University College, Aizawl, India (*vide* PUC-IAEC-2016-Z2 of 10/08/2016).

### Study period and study location

Plant specimens were collected in November 2018 from Ngopa, a village in northeast Mizoram, India. Chemical analysis and animal experiments were completed in August 2019.

### Chemicals and drug

All chemicals were analytical grades manufactured by HiMedia Laboratories Private Limited, Mumbai, India, except otherwise mentioned. Methanol for gas chromatography was a product of Merck Life Science Private Limited, Mumbai, India. Albendazole was a product of GlaxoSmithKline Pharmaceuticals Limited, Mumbai, India.

### Preparation of plant extract

*S. acmella* was collected from Ngopa, Mizoram, India, and is located between 23.8861°N and 93.2119°E. The plant specimen was identified and authenticated at the Botanical Survey of India, Shillong, India (no. PUC-A-17-1). The aerial parts of the plant were washed with distilled water and dried in shade under room temperature (23-27°C). The aqueous extract was prepared in a 5 L Soxhlet apparatus. The slurry of the extract was concentrated by removing and recovering the solvent in a vacuum rotary evaporator (Buchi Rotavapor^®^ R-215).

### Fractionation and thin-layer chromatography (TLC)

The plant extract was fractionated with methanol as an eluent in a 60 cm glass column packed with silica gel 60 (pore size 60 Å and mesh size 60-120 μm, Merck, India). TLC was performed with Merck TLC plates in aluminum oxides with 60 Å and 150 Å pore sizes and with fluorescent indicator F254 was used. Hexane + ethyl acetate was used as the solvent mixture. Colored spots observed under UV light (254 and 366 nm) were used to estimate the R_f_ values.

### Chemical analysis using gas chromatography–mass spectrometry (GC–MS)

The plant extract was analyzed in a single quadrupole gas chromatography–mass spectrometry system (Thermo Scientific TRACE™ 1300 ISQ™ LT). Methanol was used as a solvent. A non-polar column TR-5MS (260F142P, dimension of 30 m × 0.25 mm × 0.25 µm with film thickness of 0.25 μm) was used as the stationary phase. The injector port was set at 250°C. The oven was initially set at 70°C and incrementally increased to 250°C. Helium was released at 1 mL/min into the oven chamber. One microliter of the sample was injected in split mode at the splitting ratio of 1:50. The mass spectrometer was set at an ionization electron energy of 70 eV. Ion source and transfer line were set at 250°C. The total running duration was 55 min. The final chromatogram and mass spectra were generated with Thermo Scientific™ Xcalibur™ software. Compounds were identified based on their retention time, chemical formula, and molecular weight from the libraries of Wiley Registry™ 10 and National Institute of Standards and Technology database.

### Helminth survival test

The efficacy of the plant extract was tested against an intestinal cestode, *Raillietina tetragona*, Molin, 1858. The cestodes were collected from local fowl, *Gallus gallus domesticus*, Linnaeus, 1857. Concentrations of 1.25, 2.5, 5, 10, and 20 mg/ml of the plants extract were prepared by dissolving them in 0.9% neutral phosphate-buffered saline (PBS) supplemented with 1% dimethyl sulfoxide (DMSO). Corresponding concentrations were also prepared for albendazole (from the manufactured dosage of 20 mg/ml) as standard references. Control media consisted only of PBS with DMSO. Batches of two worms were selected for each test, and each test was further performed in triplicate. Death was defined as complete loss of motor activity after stimulation with tepid PBS (45°C). The times of death were recorded, and data were generated as statistical means±standard deviation. The significance of the antiparasitic activity was determined using unpaired Student’s t-test, and the level of significance was considered when p<0.05.

### Scanning electron microscopy

Cestodes treated with 20 mg/ml of the plant extract were chosen for scanning electron microscopy. The cestodes were fixed in 10% cold-buffered formaldehyde (buffered with 0.1 M sodium cacodylate) at 4°C for 4 h. Secondary fixation was done with 1% osmium tetroxide (OsO_4_) using the same buffer. The fixed specimens were then dehydrated using acetone. They were treated with tetramethylsilane, Si(CH_3_)_4_ for 15 min and left to dry in air-drying chamber at 25°C. They were sputter-coated with gold in JFC-1100 (JEOL Ltd., Tokyo, Japan) ion-sputtering chamber. Finally, they were observed under a JSM-6360 scanning electron microscope (JEOL Ltd., Tokyo, Japan) at an electron accelerating voltage of 20 kV.

## Results

### Extraction and chemical analysis

**Figure-1:**
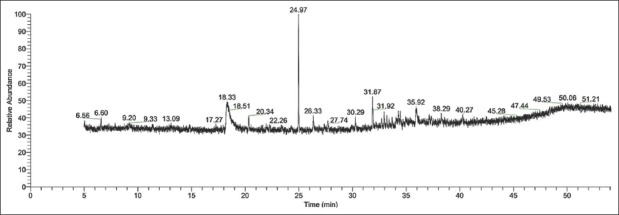
Gas chromatogram of *Spilanthes acmella* aqueous extract.

**Figure-2:**
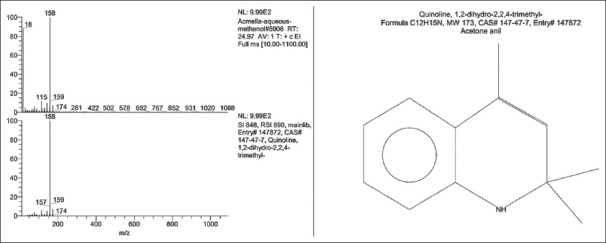
Mass spectra and chemical identity of *Spilanthes acmella* aqueous extract.

The aerial parts of *S. acmella* produced aqueous extract with an extractive value of 19.44%. The crude extract was dissolved in hexane and fractionated in a glass column. Subsequent elution was done with hexane:ethyl acetate. Each fraction was analyzed with TLC and the most bioactive fraction was found to be of aromatic compounds. Standard chemical analysis using GC–MS revealed that the extract has one major heterocyclic aromatic compound at a retention time of 24.97 min, as shown in Figure-1. Mass spectra revealed that the compound has a molecular size of 173.25 g/mol, with a relative abundance of 99.2%. From the Wiley Registry™ 10 and National Institute of Standards and Technology databases, it was confirmed that the compound has a chemical formula C_12_H_15_N, which was identified as 2,2,4-trimethyl-1,2-dihydroquinoline (Figure-2).

### Helminth survival test

The quinoline fraction of *S. acmella* extract and albendazole showed concentration-dependent activity against the cestode, *R. tetragona*. As shown in Table-1, control cestodes survived up to 74.03±1.89 h. Albendazole was highly effective in killing the cestodes in 17.59±1.43, 14.99±0.43, 12.07±0.49, 8.99±0.45, and 3.25±0.65 h at the concentrations of 1.25, 2.5. 5, 10, and 20 mg/ml (0.005, 0.009, 0.019, 0.038, and 0.075 mM), respectively. At corresponding concentrations (0.007, 0.014, 0.029, 0.058, and 0.115 mM of 2,2,4-trimethyl-1,2-dihydroquinoline), *S. acmella* extract took 48.16±1.51, 43.19±1.56, 36.32±1.00, 31.53±1.24, and 26.20±1.43 h, respectively, to completely kill the worms.

**Table-1:** Efficacy of the quinoline-rich extract of *S. acmella* and albendazole on *Raillietina tetragona*.

Treatment	Molecular concentration (mM)	Survival time (hour) in mean±SD	t value	t critical value
Control	0	74.03±1.89	NA	NA
Albendazole	0.005	17.59±1.43*	58.32	2.26
	0.009	14.99±0.43*	74.53	2.45
	0.019	12.07±0.49*	77.66	2.45
	0.038	08.99±0.45*	81.85	2.45
	0.075	03.25±0.65*	86.56	2.45
*S. acmella* extract	0.007	48.16±1.51*	26.17	2.23
	0.014	43.19±1.56*	31.57	2.26
	0.029	36.32±1.00*	43.10	2.31
	0.058	31.53±1.24*	45.97	2.26
	0.115	26.20±1.43*	49.33	2.26

* Significantly different at p<0.05 against control; NA=Not applicable; n=6; *S. acmella*=*Spilanthes acmella*

### Scanning electron microscopy

**Figure-3:**
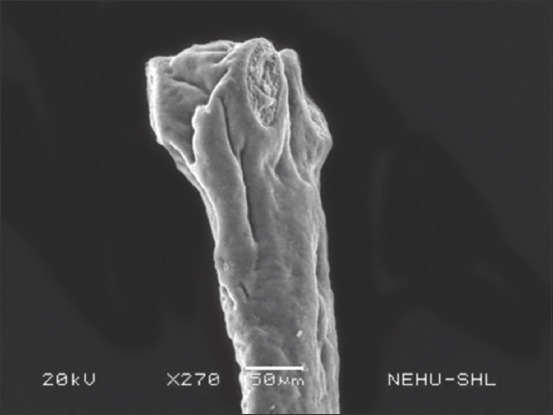
Scanning electron microscopy of the anterior portion of *Raillietina tetragona* treated with quinoline fraction of *Spilanthes acmella*. The upper tip is the scolex and the lower stalk is the neck. Three suckers can be seen on the scolex. The extreme terminal of the scolex is the rostellum, which is invaginated.

**Figure-4:**
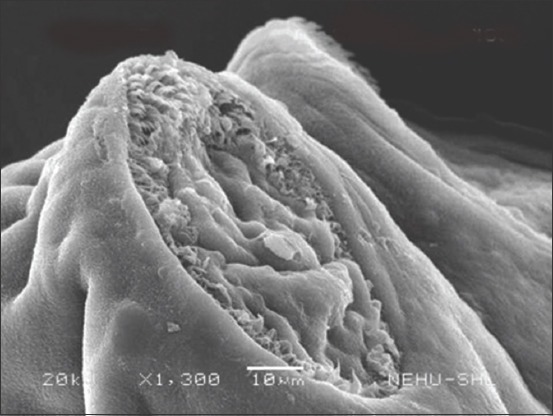
Scanning electron microscopy of the sucker region of *Raillietina tetragona* treated with quinoline fraction of *Spilanthes acmella*. Tegumental folds in the middle and loss of spines along the margin are evident. The upper rim still contains some spines, but all crooked and clumped.

**Figure-5:**
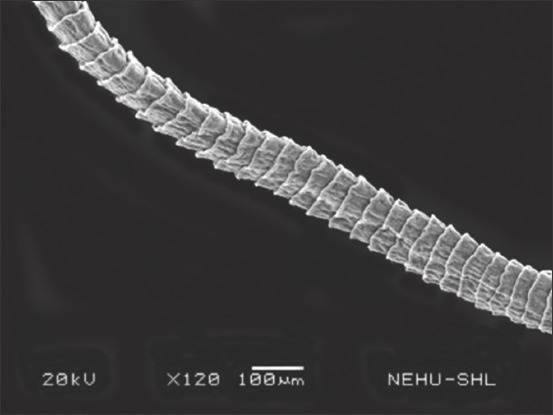
Scanning electron microscopy of the body segments (proglottids) of *Raillietina tetragona* treated with quinoline fraction of *Spilanthes acmella*. General shrinkage is seen in all the proglottids.

**Figure-6:**
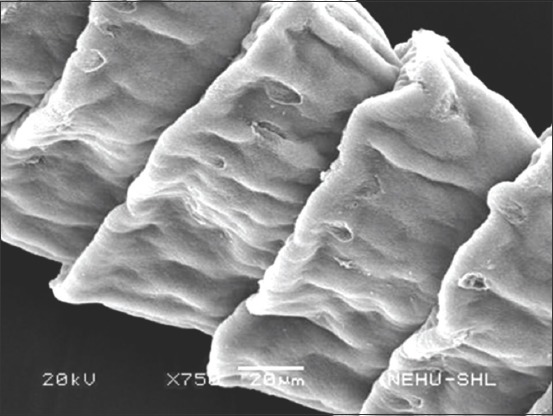
Scanning electron microscopy of the anterior proglottids of *Raillietina tetragona* treated with quinoline fraction of *Spilanthes acmella*. Mild shrinkage but massive erosions (in the form of scars) are visible in many places.

**Figure-7:**
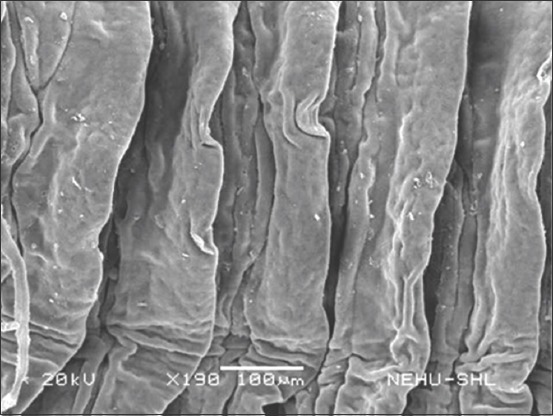
Scanning electron microscopy of the posterior proglottids of *Raillietina tetragona* treated with quinoline fraction of *Spilanthes acmella*. Shrinkage is severe, tegumental disruption is characterized by numerous pit-like scars.

Scanning electron microscopic images of *R. tetragona* after treatment with a quinoline derivative-rich extract of *S. acmella* indicate potent anthelmintic effects. The anterior portion of the body (scolex) is shown in Figures-3 and 4. Shrinkage of the body surface (tegument) is clearly evident in Figure-3. The otherwise oval-shaped and protruding suckers were distorted. The rostellum (apical end) completely collapsed and disappeared from the surface. Magnification of a single sucker (Figure-4) shows disintegration of the attachment organs (spines). The only intact spines at the top are also clumped and crooked indicating progressive disintegration. In Figure-5, general shrinkage of the body segments (proglottids) is shown. On closure examination, it is seen that shrinkage is associated with erosion of the tegument and removal of the microtriches. In the neck region, the proglottid showed relatively moderate tegumental folds, but there were massive scars (Figure-6). The mature proglottids on the posterior end of the body are severely shrunk, and tegumental erosion is indicated by the formation of tiny pit-like scars (Figure-7).

## Discussion

Historically, quinolones such as quinine from cinchona tree and synthetic chloroquine had been the mainstay of the treatment of malaria. Novel and chemical derivatives of quinolones are still considered as promising drugs for malaria [16]. Some of these novel compounds are not only effective against malarial parasites but also against other important parasites such as *Trypanosoma brucei*, *T. cruzi*, *Leishmania infantum*, and *L. amazonensis* [17]. They are also shown to have immunomodulatory, antibacterial, antifungal, antiasthmatic, antihypertensive, antitubercular, anti-inflammatory, and antiplatelet activities [18,19]. Moreover, recent studies have established that quinoline derivatives are potent inhibitors of HIV on CD4+ T cells [20].

The body surfaces of helminth parasites central to drug target because they not only serve as the immediate interface between the parasites and the host but also as the principal absorptive and sensory organs [21]. Anthelmintic drugs often destroy the parasite’s attachment organs such as suckers and rostellum. We noticed that 2,2,4-trimethyl-1,2-dihydroquinoline-rich extract of *S. acmella* induced severe structural damages on the body surface (tegument) of the parasitic cestode, *R. tetragona*. The suckers were particularly distorted with most of the spines removed or at least clumped. Albendazole reportedly caused disintegration of suckers, loss of rostellar structure, and severe tegumental contraction accompanied by erosion of microtriches in *Raillietina echinobothrida* [22]. Albendazole and flubendazole effectively induced collapse of the rostellum, defacement of the microtriches, eruption of swellings or blebs on the tegument, and increased vesiculation on the tapeworm, *Echinococcus granulosus* [23]. *Mesocestoides corti* developed crooked spines, distorted suckers, and scarring of the tegument after a combination treatment with albendazole and praziquantel [24].

The tegumental damages, including general shrinkage, destruction of sucker, erosion of spines, and microtriches on *R. tetragona*, are, therefore, in agreement with anthelmintic effects reported for different drugs on different helminth parasites. In addition, *Schistosoma mansoni* exhibited extensive disintegration, sloughing, and erosion of the tegument after treatment with praziquantel [25]. Treatment with praziquantel-resveratrol combination resulted in degeneration of the tegumental and subtegumental tissues in *S. mansoni* [26]. Praziquantel and *Carica papaya* seed extract caused shrinkage of the tegument, rostellar swelling, and complete removal of spines in *Hymenolepis nana* [27]. Piplartine, an amide from *Piper tuberculatum*, also caused extensive destruction of the tegument on the oral and ventral sucker regions of *S. mansoni* [28].

## Conclusion

We produced an extract of *S. acmella* that contained a quinoline derivative, which was identified from GC–MS data as 2,2,4-trimethyl-1,2-dihydroquinoline. This compound has no known biological effects. We showed that it was highly effective against a parasitic cestode, *R. tetragona*. Tegumental damages, loss of microtriches and disintegration of suckers of the cestode after *in vitro* treatment are sufficient evidence to infer that the quinoline derivative is an interesting antiparasitic compound.

## Authors’ Contributions

KL conceived the experimental design. PBL performed the experiments. KL analyzed the data and wrote the first draft. PBL completed the writing. Both authors have read and approved the final manuscript.
